# Renal Dysfunction in Pediatric Patients in Iraq With β-Thalassemia Major and Intermedia

**DOI:** 10.7759/cureus.29183

**Published:** 2022-09-15

**Authors:** Mohammad G Shaalan, Meaad K Hassan, Hamid J Al-Shanoof, Lamia M Al Naama

**Affiliations:** 1 Department of Pediatrics, Basrah Maternity and Child Hospital, Basrah, IRQ; 2 Department of Pediatrics, College of Medicine, University of Basra, Basrah, IRQ; 3 Department of Biochemistry, Al-Fahaa Teaching Hospital/ Al-Zehraa Medical College, Basrah, IRQ; 4 Department of Biochemistry, College of Medicine, University of Basra, Basrah, IRQ

**Keywords:** urine ratio, children, iron overload, renal function, β-thalassemia

## Abstract

Background

With optimum transfusion and chelation therapy, the survival of β-thalassemia patients and the incidence of various complications, including renal complications, have improved.

Objectives

To investigate renal involvement in β-thalassemia patients using serum and urinary biochemical markers of glomerular and tubular dysfunction.

Methods

This case-control study included 69 β-thalassemia major (β-TM) patients, 23 β-thalassemia intermedia (β-TI) patients, and 100 healthy controls, all ranging from 1 to 16 years in age. Blood urea nitrogen (BUN), serum ferritin, serum and urinary levels of creatinine (Cr), uric acid (UA), calcium (Ca), phosphorus (Ph), magnesium (Mg), sodium (Na), and potassium (K), and the urinary albumin/creatinine ratio were evaluated.

Results

The BUN level and the urinary Na/Cr, K/Cr, Ca/Cr, Mg/Cr, Ph/Cr, albumin/Cr, and UA/Cr ratios were significantly higher in the β-thalassemia patients than in the controls. In contrast, the serum Na, K, Ca, and Mg levels were significantly lower in the patients (P<0.05). An elevated urinary UA/Cr ratio was found in 61.9% of β-thalassemia patients, and an elevated urinary Ca/Cr, and urinary albumin/Cr ratio was found in 53.2%. An elevated Na/Cr ratio was found in 41.3%. The serum and urinary renal markers showed no significant differences between patients with β-TM and β-TI, except for microscopic hematuria, which was significantly higher in β-TI patients (34.8%) than in β-TM patients (13%), P>0.02. At an older age, high serum ferritin levels and deferoxamine therapy were associated with significant tubular and glomerular dysfunction in β-thalassemia patients.

Conclusions

Pediatric patients with β-thalassemia have significantly abnormal tubular and glomerular functions, necessitating early detection and monitoring to prevent/reverse renal function deterioration.

## Introduction

Beta-thalassemia is a group of hereditary blood disorders characterized by the reduction or absence in the synthesis of the β chains of hemoglobin, resulting in variable phenotypes ranging from no clinical symptoms to severe anemia [1]. Patients with a severe form of β-thalassemia, such as β-thalassemia major (β-TM), present early in infancy with debilitating anemia that requires life-long regular transfusions for survival. However, patients with β-thalassemia intermedia (β-TI) present with less severe anemia later in life and remain transfusion independent except within specific clinical settings [2]. Although the survival of patients with β-thalassemia has improved because of blood transfusion, iron chelation therapy, and advances in knowledge of the disease, many patients develop various complications, including cardiopulmonary complications, endocrine diseases, liver impairment, and thromboembolism [3,4]. Among patients with β-TM, a shortened RBC life span, hypoxia, rapid iron turnover, excess iron deposition in tissue, and the use of specific iron chelators may adversely affect the kidneys [5-7]. Splenectomy has also been associated with renal complications, including tubular and glomerular dysfunction, in these patients [8,9]. In β-TI, iron overload (IOL) occurs in non-transfused patients because of increased intestinal iron absorption due to ineffective erythropoiesis, which can eventually cause complications similar to those observed in β-TM patients [3]. Renal disease is considered the fourth most common cause of morbidity in patients with β-TM. Renal disease may present in many forms, such as renal tubular acidosis, glomerular dysfunction, urine concentration failure, proteinuria, aminoaciduria, renal tubular damage and excess secretion of proximal tubule damage markers, such as N-acetyl-beta-D-glucosaminidase (NAG). Thus, the early identification of patients at risk of developing renal impairment is essential to prevent and/or reverse the deterioration in renal function, improving the prognosis and reducing the incidence of end-stage renal failure and mortality [7,10]. Our objectives were to investigate the presence of glomerular and/or tubular dysfunction in children and adolescents with β-TM and β-TI using both serum and urinary biochemical variables and to correlate the renal findings to age, serum ferritin, and iron chelation therapy.

## Materials and methods

A case-control study was carried out in known β-thalassemia patients (β-TM and β-TI) registered at the Basrah Center of Hereditary Blood Diseases. These patients ranged in age from 1 to 16 years. The control group included age- and sex-matched healthy children randomly selected from among children consulting the outpatient department for minor illnesses (such as upper respiratory tract infection) or the dental clinic.

Data collection

Complete clinical data were obtained from β-thalassemia patients, including the age at diagnosis, age at presentation, sex, number and frequency of blood transfusions, urinary symptoms, and treatment with iron-chelating agents. In addition, the diagnosis concerning the type of thalassemia, i.e., β-TM or β-TI, was recorded for all patients. Children and adolescents with β-thalassemia known to have a history of renal pathology, such as nephrotic syndrome, systemic illnesses (cardiac complications, thyroid diseases, hepatic diseases or diabetes mellitus), on diuretic therapy, or to have a history of the intake of nephrotoxic drugs, such as corticosteroids, trimethoprim and cephalosporin, in the last week were excluded from this study [10]. Informed consent was obtained from one of the parents of each participant before recruitment into the study. The Ethical Committee of Basra Medical College approved the study.

Methods 

Venous blood was collected from the patients and controls under standardized conditions. A portion of each blood sample was added to ethylenediamine tetraacetic acid (EDTA) tubes for hemoglobin (Hb) analysis, and the remainder was transferred to plain tubes for the rest of the biochemical analyses. After sera separation, specimens were either immediately analyzed or stored in freezing conditions until analysis within two days. The Hb level was measured using the Sysmex™ Automated Hematology Analyzer KX-21N (Sysmex Corporation, Kobe, Japan). The biochemical variables: blood urea nitrogen (BUN), serum creatinine (Cr), serum uric acid (UA), serum phosphorus (Ph), serum magnesium (Mg), and serum calcium (Ca) levels were measured by the Automated Clinical Chemistry Analyzer; Cobas INTEGRA 400 plus (Roche, Germany). Both the serum sodium (Na) and potassium (K) levels were estimated by flame photometry. Furthermore, the serum ferritin (mg/l) level was assessed on a MINI VIDAS system using bioMérieux kits (France). Urine samples were obtained from first-morning urination specimens while patients and controls were afebrile. First, the urinary RBC and WBC were assessed either microscopically or by urinary dipstick. Thereafter, the urine samples were stored at -4C° until analysis to estimate the urinary parameters. The urinary Ca, Mg, Ph, UA, and Cr levels were estimated using the same methods as the serum levels. In contrast, the urinary albumin, Cr, Na, and K levels were measured using the Automated Clinical Chemistry Analyzer ARCHITECT plus c4000 (Abbott Laboratories, USA). All the procedures were followed as described by the manufacturer. The urinary Na/Cr, K/Cr, Ca/Cr, Mg/Cr, Ph/Cr, UA/Cr, and urinary albumin/Cr ratios were then calculated for each subject (patients and controls) by dividing each urinary variable by its urine Cr value. These urinary variables were taken as ratios because it was difficult to get 24-hour urine collection from these individuals. Renal USG was performed for all patients enrolled in the study to detect the presence of renal stones and the size of the kidneys. 

Definition of variables

The normal values of the biochemical variables were determined according to levels measured in the healthy children and adolescents recruited in this study and are presented as the means ± SDs. All biochemical test results are stratified as low (defined as a level > 2 SD below the mean), normal (a level within 2 SD of the mean), or high (a level >2 SD above the mean). The serum ferritin levels in all patients with β-TM and β-TI were divided into three groups: those with serum ferritin <1000 ng/ml, between 1000 and 3000 ng/ml, and >3000 ng/ml. Hematuria was defined as the presence of >5 RBCs/high power field (HPF) in a centrifuged urine specimen [11]. Microalbuminuria was defined as urinary excretion of albumin/Cr ratio in the range of 30-300 mg/g Cr, while macroalbuminuria was defined as albumin/Cr ratio >300mg/g Cr [12]. Depending on these laboratory investigations, renal tubular dysfunction can be differentiated from glomerular dysfunction, as renal tubular dysfunction is characterized by elevated urinary Ca/Cr, Ph/Cr, Mg/Cr, and UA/Cr ratios. In contrast, glomerular dysfunction is characterized by an elevated urinary RBC level, albumin/Cr ratio (>300 mg/g), and serum Cr level. In addition, other variables are affected in both tubular and glomerular dysfunction [9,10,12].

Statistical analysis

Statistical analysis was performed using SPSS software version 17 (IBM, Chicago, Illinois, USA). Data are presented as the mean ± SD or number and percentage (N and %), as appropriate, and as tables or figures. Comparisons of proportions were performed with crosstabs by Chi-square and Fisher's exact test. Statistical comparisons of means were performed by t-test and ANOVA. For all tests, a p-value <0.05 was considered statistically significant. 

## Results

A total of 92 children and adolescents with β-thalassemia were included in the study: 69 with β-TM and 23 with β-TI. Their ages ranged from 12 months to 16 years, with a mean age of 7.76 ± 3.46 years. Forty-four patients (47.8%) were males, and 48 (52.2%) were females. The control group included 100 age-matched children and adolescents, and their mean age was 8.15 ± 3.77 years; 50 (50%) were males, and 50 (50%) were females, and there was no significant difference in age or sex between the patients and controls (P>0.05). Although evaluation of the different renal markers revealed normal mean levels for almost all studied variables, the BUN level was significantly higher, while the Hb, serum Na, K, Ca, and Mg levels were significantly lower in β-thalassemia patients than in the controls (P<0.05, Table 1).

**Table 1:** Hemoglobin level, serum and urinary biochemical variables in β-thalassemia patients and the controls. Independent t-tests were used to assess p-values. Abbreviations: S: Serum; BUN: Blood urea nitrogen; Cr: Creatinine; Na: Sodium; K: Potassium; Ca: Calcium; Mg: Magnesium; Ph: Phosphorus; UA: Uric acid.

Variables	Patients (Mean±SD, N=92)	Controls (Mean ±SD, N=100)	P-value	
				Blood Parameters				
Hemoglobin (gm/dl)	6.86±2.00	11.8±2.10	0.001	
BUN (mg/dl)	18.7±2.99	12.86±3.28	<0.001	
S Cr (mg/dl)	1.19±0.39	1.09±0.29	0.053	
S Na (mEq/l)	138.44±7.2	143.01±5.28	<0.001	
S K (mEq/l)	4.01±0.50	4.22±0.48	0.003	
S Ca (mg/dl)	8.65±0.56	9.35±0.72	<0.001	
S Mg (mg/dl)	1.84±0.15	1.91±0.15	0.010	
S Ph (mg/dl)	4.70±0.55	4.65±0.45	0.782	
S UA (mg/dl)	3.71±1.11	4.02± 0.64	0.050	
Urinary Parameters				
Na/Cr ratio	2.19±1.63	1.43±1.13	<0.001	
K/Cr ratio	0.95±0.60	0.65±0.39	<0.001	
Ca/Cr ratio	0.18±0.04	0.15±0.03	<0.001	
Mg/Cr ratio	0.16±0.03	0.13±0.02	<0.001	
Ph/Cr ratio	0.56±0.15	0.42±0.07	<0.001	
UA/Cr ratio	0.88±0.16	0.40±0.06	0.013	
Albumin/Cr ratio	61.13±17.47	22.4±7.35	0.006	
*Urine RBCs > 5/HPF	17 (18.4%)	0 (0)		

Furthermore, the urinary Na/Cr, K/Cr, Ca/Cr, Mg/Cr, Ph/Cr, UA/Cr, and albumin/Cr ratios were significantly higher in the β-thalassemia patients than in the controls (P<0.05). None of the patients or controls showed pyuria or other urinary changes except for microscopic hematuria, which was present in 18.4% of patients and none of the controls. The mean age at diagnosis was 10.48±4.35 months in β-TM patients compared to 39.13±7.71 months in β-TI patients (P<0.001). Loin pain and dysuria were the main renal manifestations, each reported in 10 (10.86%) patients. No patient had edema, polyuria, nocturia, frequency, or fever at the time of the study (Table 2).

All patients recruited in the study had a history of blood transfusion; however, the number of blood transfusions per year was significantly higher in patients with β-TM (P<0.001). There was no significant difference in the frequency of splenectomy between the patient groups. The serum and urinary renal markers were not significantly different between patients with β-TM and β-TI (P>0.05); except for the Hb level that was significantly lower in patients with β-TM (P=0.001, Table 2),

**Table 2:** Selected clinical, serum and urinary biochemical variables in patients with β-TM and β-TI. Independent t-tests were used to assess p-values based on the mean ± SD of the two groups. * Values are expressed as N. (%), Chi-square tests or Fisher's exact test were used to assess p-values. Abbreviations: β-TM: Beta-thalassemia major; β-TI: Beta-thalassemia intermedia; : S: Serum; BUN: Blood urea nitrogen; Cr: Creatinine; Na: Sodium; K: Potassium; Ca: Calcium; Mg: Magnesium; Ph: Phosphorus; UA: Uric acid.

Variables		β-TM (Mean±SD, N=69)	β-TI (Mean±SD, N=23)	P-value
Clinical variables				
Age at DX (months)		10.48±4.35	39.13±7.71	<0.001
Dysuria N. (%)		5 (7.2)	5 (21.7)	0.066*
Loin pain N. (%)		6 (8.7)	4 (17.3)	0.214*
No. BT/year N. (%)	< 12	2 (2.9)	17 (73.9)	<0.001*
	> 12	67 (97.1)	6 (26.1)	
Splenectomy N. (%)		23 (33.3)	8 (34.7)	0.494*
Laboratory variables A. Blood parameters				
Hemoglobin (gm/dl)		6.1±1.45	9.41±1.02	0.001
BUN (mg/dl)		18.73±3.46	18.36±2.95	0.411
S Cr (mg/dl)		1.21±0.39	1.18±0.30	0.561
S Na (mEq/l)		137.42±7.4	140.9±7.7	0.425
S K (mEq/l)		3.88±0.49	4.17±0.52	0.365
S Ca (mg/dl)		8.63±0.68	8.70±0.55	0.329
S Mg (mg/dl)		1.84±0.15	1.84±0.15	0.239
S Ph (mg/dl)		4.70±0.51	4.68±0.58	0.419
S UA (mg/dl)		3.69±1.10	3.75±1.00	0.224
B. Urinary parameters				
Na/Cr ratio		2.36±1.76	2.11±1.4	0.540
K/Cr ratio		0.94±0.54	0.97±0.74	0.800
Ca/Cr ratio		0.19±0.04	0.18±0.04	0.639
Mg/Cr ratio		0.17±0.04	0.15±0.03	0.254
Ph/Cr ratio		0.56±0.16	0.57±0.14	0.393
UA/Cr ratio		0.65±0.17	0.65±0.18	0.934
Albumin/Cr ratio		67.54±12.57	53.53±18.90	0.084
*Urine RBCs > 5/HPF		9 (13%)	8 (34.8%)	0.026

**Figure 1:**
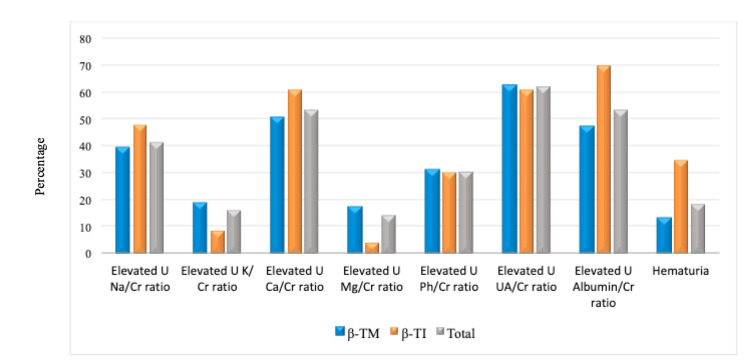
Main glomerular and tubular abnormalities in patients with β-thalassemia major and intermedia. Abbreviations: S: Serum; BUN: Blood urea nitrogen; Cr: Creatinine; Na: Sodium; K: Potassium; Ca: Calcium; Mg: Magnesium; Ph: Phosphorus; UA: Uric acid.

The urinary UA/Cr ratio was the most common renal tubular function marker affected in β-thalassemia patients (61.9%), followed by the urinary Ca/Cr ratio (53.2%), Na/Cr ratio (41.3%), Ph/Cr ratio (30.4%), K/Cr ratio (16.3%), and Mg/Cr ratio (14.2%), with no significant difference in the frequency of these abnormalities between β-TM and β-TI patients (P>0.05). However, the urinary albumin/Cr ratio, as an indicator of renal glomerular function, was affected in 53.2% of β-thalassemia patients (47%, β-TM; 70%, β-TI, P=0.084), and the serum Cr level was elevated in 23.9% of patients (27%, β-TM; 17.4%, β-TI, P>0.05). Microscopic hematuria was also more common in β-TI (34.8%) than β-TM (13%) patients (P=0.026, Figure 1).

According to age, the patients with β-thalassemia were divided into three subgroups, i.e., those ≤5 years of age, 6-10 years of age, and >10 years of age. Both the BUN and serum Cr levels were significantly higher in patients older than 10 years, while the serum Ca level was significantly lower in patients older than 10 years (P<0.05). In addition, the urinary Ca/Cr, Ph/Cr, and albumin/Cr ratios were significantly higher in the older patients (P<0.05, Table 3).

**Table 3:** Serum and urinary biochemical variables in relation to patient's age. ANOVA was used: ^a ^P-value (<0.05) between groups I and III, ^b ^P-value (<0.05) between groups I and II, ^c ^P-value (<0.05) between groups II and III. Abbreviations: S: Serum; BUN: Blood urea nitrogen; Cr: Creatinine; Na: Sodium; K: Potassium; Ca: Calcium; Mg: Magnesium; Ph: Phosphorus; UA: Uric acid.

Variables	Patient age (year)			P-value
	Group I ≤5 (Mean±SD, N=25)	Group II 6-10 (Mean±SD, N=47)	Group III >10 (Mean±SD, N=20)	
Blood Parameters				
BUN (mg/dl)	17.43±3.27	18.37±2.12	19.37±3.92	0.025^a^
S Cr (mg/dl)	1.21±0.44	1.26±0.47	1.34±0.41	0.034^a^
S Na (mEq/l)	140.35±6.55	136.36±8.24	137.5±5.1	0.253
S K (mEq/l)	4.03±0.40	4.25±0.55	4.07±0.55	0.516
S Ca (mg/dl)	9.58±0.62	8.65±0.69	8.58±0.66	0.044^a^
S Mg (mg/dl)	1.89±0.13	1.84±0.15	1.87±0.18	0.437
S Ph (mg/dl)	4.69±0.55	4.76±0.46	4.55±0.60	0.095
S UA (mg/dl)	3.98±1.27	3.81±0.89	4.09±0.90	0.300
Urinary Parameters				
Na/Cr ratio	2.92±1.41	2.56±1.63	2.55±1.74	0.155
K/Cr ratio	0.96±0.62	0.94±0.9	0.95±0.62	0.082
Ca/Cr ratio	0.18±0.03	0.19±0.04	0.20±0.03	0.003^c^
Mg/Cr ratio	0.16±0.03	0.15±0.03	0.15±0.04	0.523
Ph/Cr ratio	0.52±0.16	0.56±0.13	0.63±0.17	0.023^a^
UA/Cr ratio	0.68±0.19	0.62±0.16	0.69±0.18	0.153
Albumin/Cr ratio	32.40±12.34	43.64±17.84	140.6±132.4	<0.05^a,c^

Other findings of the study include a significant increase in the BUN, serum Cr and serum Mg levels with increasing serum ferritin levels. In contrast, the serum Ca level decreased significantly with increasing serum ferritin levels (P<0.05). In addition, the urinary albumin/Cr, Ph/Cr, Ca/Cr and Mg/Cr, and UA/Cr ratios were significantly higher in patients with serum ferritin levels >3000 ng/dl than in those with lower levels (P<0.05, Table 4).

**Table 4:** Serum and urinary biochemical variables in relation to the serum ferritin level. ANOVA was used. ^a^: P-value (<0.05) between groups I and III, ^b^:P-value (<0.05) between groups I and II, ^c:^P-value (<0.05) between groups II and III. Abbreviations: S: Serum; BUN: Blood urea nitrogen; Cr: Creatinine; Na: Sodium; K: Potassium; Ca: Calcium; Mg: Magnesium; Ph: Phosphorus; UA: Uric acid.

Variables	S Ferritin (ng/ml)			P-value
	Group І ≤1000 (Mean±SD, N= 23)	Group ІI >1000-3000 (Mean±SD, N=34)	Group III >3000 (Mean±SD, N=35)	
Blood Parameters				
BUN (mg/dl)	17.41±3.3	18.13±3.2	18.82±3.4	0.044^a^
S Cr (mg/dl)	1.30±0.50	1.34±0.32	1.35±0.37	0.046^a^
S Na (mEq/l)	140.37±6.92	138.24±8.31	138.03±7.51	0.340
S K (mEq/l)	3.78±0.43	4.12±0.51	3.91±0.51	0.080
S Ca (mg/dl)	9.05±0.63	8.62±0.63	8.60±0.65	0.040^a^
S Mg (mg/dl)	1.83±0.11	1.80±0.16	1.86±0.15	0.014^c^
S Ph (mg/dl)	4.74±0.50	4.59±0.42	4.74±0.57	0.053^b^
S UA (mg/dl)	3.74±0.95	3.86±1.08	3.64±1.10	0.561
Urinary Parameters				
Na/Cr ratio	2.18±1.41	1.82±1.60	2.18±1.57	0.561
K/Cr ratio	0.90±0.40	0.91±0.38	0.94±0.41	0.493
Ca/Cr ratio	0.18±0.04	0.19±0.03	0.21±0.04	0.048^a^
Mg/Cr ratio	0.16±0.04	0.16±0.04	0.19±0.04	0.042^a^
Ph/Cr ratio	0.42±0.07	0.49±0.12	0.60±0.16	<0.01^a,c^
UA/Cr ratio	0.59±0.23	0.55±0.16	0.63±0.17	<0.01^a,c^
Albumin/Cr ratio	37.72±10.92	37.27±15.06	83.91±89.16	<0.05^a,c^

The role of iron chelation therapy, i.e., deferoxamine (DFO), was also investigated. The mean BUN level was significantly higher, while the serum Ca and UA levels were significantly lower in patients treated with DFO than those not treated with DFO (P<0.05). In addition, the urinary Ca/Cr, UA/Cr, and albumin/Cr ratios were significantly higher in patients treated with DFO than those not treated with DFO (P<0.05, Table 5).

**Table 5:** Serum and urinary biochemical variables in relation to iron chelation therapy. Independent t-tests were used to assess p-values. Abbreviations: S: Serum; BUN: Blood urea nitrogen; Cr: Creatinine; Na: Sodium; K: Potassium; Ca: Calcium; Mg: Magnesium; Ph: Phosphorus; UA: Uric acid.

Variables	Patient group		P-value
	With DFO (Mean±SD, N=48)	Without DFO (Mean±SD, N=44)	
Blood Parameters			
BUN (mg/dl)	19.45 ± 3.32	17.73 ± 3.13	0.015
S Cr (mg/dl)	1.21±0.4	1.18±0.30	0.700
S Na (mEq/l)	136.82±7.46	138.11±6.49	0.385
S K (mEq/l)	3.93±0.52	3.82±0.46	0.079
S Ca (mg/dl)	8.53±0.61	8.90±0.55	0.031
S Mg (mg/dl)	1.84±0.17	1.84±0.14	0.061
S Ph (mg/dl)	4.65±0.57	4.74±0.48	0.208
S UA (mg/dl)	3.69±1.24	3.86±0.89	0.040
Urinary Parameters			
Na/Cr ratio	2.55±1.68	2.54±1.58	0.150
K/Cr ratio	0.95±0.60	0.96±0.90	0.125
Ca/ratio	0.21±0.04	0.18±0.04	0.011
Mg/Cr ratio	0.17±0.04	0.15±0.03	0.387
Ph/Cr ratio	0.56±0.15	0.58±0.16	0.141
UA/Cr ratio	0.71±0.15	0.59±0.21	0.045
Albumin/Cr ratio	81.5±15.1	43.13±27.71	0.042

An abdominal ultrasound (US) was performed for all patients recruited in the study; eight (11.5%) patients with β-TM and five (21.7%) patients with β-TI had renal stones, while others had normal USG findings. Among the patients with renal stones, 10 (76.9%) had loin pain.

## Discussion

With the improved survival of patients with β-thalassemia, evaluation of the kidneys (in terms of both tubular and glomerular function) has become more common to identify previously unrecognized complications of the disease and/or facilitate its treatment.

In this case-control study, clinical and laboratory findings were used to assess renal dysfunction in patients with β-TM and β-TI. The study revealed that children and adolescents with β-thalassemia have abnormal renal tubular and glomerular function, as indicated by higher urinary Na/Cr, K/Cr, Ca/Cr, Mg/Cr, Ph/Cr, UA/Cr, and albumin/Cr ratios in the patients than in the controls. However, the serum and urinary renal markers were not found to be significantly different between patients with β-TM and β-TI.

Loin pain and dysuria were the main clinical manifestations of renal involvement among patients with β-TM and β-TI. The loin pain in the current study was most likely caused by renal stones (76.9%), as detected by the abdominal US. Among patients with dysuria, none of these patients or others were confirmed to have pyuria on urinary dipstick testing. Dysuria, in the absence of pyuria, in thalassemia patients in the current study can be explained by hypercalciuria, which predisposes patients to urinary symptoms, including dysuria [13].

Our results differ from those of Ali D et al., who reported that pyuria was the primary renal manifestation, occurring in 12% of patients with β-TI and 5% of patients with β-TM, followed by renal stones [14]. However, Fallahzadeh MK et al. reported that hematuria and pyuria were the most common renal manifestations of glomerular dysfunction in β-TM [15].

In the current study, microscopic hematuria was reported in 18.5% of total cases, with 13% of patients with β-TM and 34.8% of patients with β-TI. The overall frequency is comparable to that reported by Ali D et al. (17.6%), with a higher frequency in β-TI [14]. Furthermore, Fallahzadeh MK et al. reported microscopic hematuria in 10.6% of β-TM patients [15]. The presence of hematuria may be related to either hypercalciuria or hyperuricosuria and an increased incidence of renal stones [7,14].

The serum Cr and electrolyte levels were within normal limits in our β-thalassemia patients; however, the mean levels of serum Na, K, Ca, and Mg were significantly lower in the patients than in the controls. While the urinary Na/Cr, K/Cr, Ca/Cr, Mg/Cr, Ph/Cr, UA/Cr, and albumin/Cr ratios were significantly higher in the β-thalassemia patients than in the controls.

Different researchers have reported conflicting results. For example, Mahmoud AA et al. reported a significant increase in serum urea, Cr, and K in β-TM patients compared with the controls but no significant difference in serum Na and Ca between the two groups. In addition, they found a significant increase in urinary UA/Cr, U Ca/Cr, U albumin/Cr, and UK/Cr in β-TM patients than in the controls [16].

Şen V et al. also did not find a significant difference in the serum urea, Cr, or serum and urinary electrolyte (Na, K, Ca, and Ph) levels between patients and controls, aside from a higher urinary protein/Cr ratio in thalassemia patients [17]. At the same time, Hamed EA et al. reported that the serum Cr, serum phosphate, and serum UA levels and the urinary albumin/Cr, Ca/Cr, UA/Cr, and Ph/Cr ratios were significantly higher in the β-thalassemia patients than in the controls [10].

The increased urinary excretion of UA and electrolytes in β-thalassemia can be explained by chronic anemia/hypoxia with rapid RBC turnover, IOL, and nephrotoxic iron chelators leading to decreased renal tubular reabsorption and increased loss in urine [4,7,18].

In our study, apart from microscopic hematuria, no significant differences in renal findings were found between patients with β-TM and β-TI. These findings are in agreement with that of Uzun E et al. in Turkey [19]. Adly AA et al. reported a significant difference in the serum Na, K, and Ph levels but no significant difference in the urinary excretion of these electrolytes between the two types [20]. Ali D et al. reported that children with β-TM had significantly higher serum Cr and blood urea levels and lower serum UA levels than β-TI patients [14].

In this study, the frequency of tubular dysfunction was higher than that of glomerular dysfunction according to the frequency of hyperuricosuria compared to that of microalbuminuria, and these results are similar to those described by Economou M et al.. They reported that 60% of patients with β-TM had renal tubular dysfunction [21]. On the other hand, Ponticelli C et al. reported nearly equal frequencies of tubular and glomerular dysfunction in β-TM, along with the frequency of hyperuricosuria (38%) and an impaired glomerular filtration rate (GFR, 40%) [5]. Renal tubular and glomerular dysfunctions are not uncommon in patients with β-TM and β-TI. IOL, anemia, and iron chelation therapy are likely to be the main factors responsible for these tubular and glomerular abnormalities. IOL leads to iron deposition in the glomeruli, proximal renal tubules, and renal interstitium. Anemia causes glomerular hyperperfusion, and hyperfiltration can lead to stretching of the glomerular capillary wall, resulting in endothelial and epithelial injury and hypoxia, which in turn leads to tubule-interstitial injury [4,5,7]. In this study, the effect of age on renal function was evaluated. The results of this study reveal that the BUN and serum Cr levels and the urinary Ca/Cr, albumin/Cr, and urinary Ph/Cr ratios were significantly higher, while the serum Ca level was significantly lower in older patients aged more than 10 years as compared to younger one aged less than 10 years. The impact of age on renal function in β-thalassemia is consistent with that reported by other researchers [10,15], probably due to the increased duration of blood transfusion, IOL, and iron chelation therapy in these patients.

In β-thalassemia, IOL is associated with increased morbidity in both patients with transfusion-dependent thalassemia and non-transfusion-dependent thalassemia, including the morbidity affecting the liver, heart, endocrine system, and kidneys [22,23]. The serum ferritin level is the most commonly used marker to test for IOL in β-TM, with target ferritin of approximately 1000 mg/l, as it correlates with body iron stores and is relatively easy and inexpensive to perform [24,25]. However, serum ferritin is less reliable in β-TI, although it can be used for monitoring IOL when other modalities to assess IOL are unavailable [26], as in Iraq. The current study demonstrates both abnormal glomerular and tubular markers in patients with severe IOL. Other studies from different countries have reported similar results [14,27]. The mechanism of IOL-associated renal damage has not been fully explained. Non-transferrin-bound iron can lead to organelle membrane dysfunction and subsequent cell injury/death. Chronic iron deposition in proximal tubules, glomeruli, and the interstitium can lead to glomerulo-sclerosis, tubular atrophy, and interstitial fibrosis [4,7].

Iron chelators (DFO and deferasirox [DFX]) may also affect the renal function of patients with β-thalassemia, although renal manifestations attributed to chelating agents are rare [7]. Both DFO and DFX can cause increases in serum Cr, proteinuria, and even renal failure [5,10,28]. The results of this study reveal that the BUN level and urinary Ca/Cr, UA/Cr, and albumin/Cr ratios were significantly higher, while the serum Ca and UA levels were significantly lower in patients on DFO therapy.

Economou M et al. reported the rates of impaired renal function with elevated cystatin C levels (36%), glomerular dysfunction with proteinuria (24%), and tubulopathy with hypercalciuria (35.5%), among children and adolescents with β-TM on iron chelation therapy [21]. Hamed EA et al. reported that β-TM patients on DFO therapy showed an impaired GFR (58.82%), microalbuminuria (47.10%), and elevated serum Cr levels and UA/Cr ratios, suggesting a nephrotoxic effect of DFO [10].

Limitations of the study

The current study has many limitations. First, this was a cross-sectional study, and to monitor changes in renal function over time, it is better to perform longitudinal studies. Second, other sensitive markers for monitoring glomerular and tubular dysfunction, such as the cystatin C level and beta 2 microglobulin/Cr ratio, were not evaluated. Another limitation is that the effect of deferasirox (DFX) was not studied due to the limited number of patients receiving this drug at the time of the study. Therefore, the nephrotoxic effects of this iron chelator, DFX, were not assessed.

## Conclusions

Asymptomatic renal tubular and glomerular dysfunctions are not uncommon in pediatric patients with β-TM and β-TI. These renal complications increase with the patient's age, IOL, and DFO therapy. Microscopic hematuria is significantly more common in β-TI than in β-TM. Therefore, the routine monitoring of renal function in all patients with β-thalassemia is recommended, and further studies are required to evaluate these findings and their long-term effects on patients with β-thalassemia.
